# Prox1 and FOXC2 Act as Regulators of Lymphangiogenesis and Angiogenesis in Oral Squamous Cell Carcinoma

**DOI:** 10.1371/journal.pone.0092534

**Published:** 2014-03-19

**Authors:** Tomonori Sasahira, Nobuhiro Ueda, Kazuhiko Yamamoto, Miyako Kurihara, Sayako Matsushima, Ujjal K. Bhawal, Tadaaki Kirita, Hiroki Kuniyasu

**Affiliations:** 1 Department of Molecular Pathology, Nara Medical University, Kashihara, Japan; 2 Department of Oral and Maxillofacial Surgery, Nara Medical University, Kashihara, Japan; 3 Department of Biochemistry and Molecular Biology, Nihon University School of Dentistry at Matsudo, Matsudo, Japan; University of Nebraska Medical Center, United States of America

## Abstract

Prospero homeobox 1 (Prox1) and forkhead box (FOX) C2 regulate angiogenesis and/or lymphangiogenesis. However, the detailed role and function of Prox1 and FOXC2 in cancer remains controversial. In the present study, we examined the expression of Prox1 and FOXC2 proteins in specimens from 163 cases with oral squamous cell carcinoma (OSCC). Furthermore, the role of Prox1 and FOXC2 in cancer cell growth and invasion was evaluated in cultured OSCC cells. Prox1 expression was significantly associated with local progression of the tumor (P = 0.0023), clinical stage (P<0.0001), lymphovessel density (LVD) (P<0.0001), nodal metastasis (P<0.0001), and worse prognosis (P<0.0001). Immunoreactivity of FOXC2 was strongly correlated with microvessel density (MVD) (P<0.0001) and poor prognosis (P = 0.0076). *In vitro* analysis demonstrated that Prox1 regulates cell growth, proliferation, invasion, and lymphangiogenesis by activating vascular endothelial growth factor (*VEGF*)*-C* expression. Furthermore, FOXC2 enhanced the expression level of *Prox1* and promoted angiogenesis by enhancement of *VEGF-A* expression. Our results suggested that Prox1 and FOXC2 play key roles in OSCC progression and that further studies focusing on these proteins may yield useful insights for diagnosis and therapy of OSCC.

## Introduction

Head and neck cancers, including oral squamous cell carcinoma (OSCC), are the sixth most common malignancy in the world [1] and the first leading cause of cancer death in Southern Asia [2]. Every year, 263,900 cases of OSCC and 128,000 OSCC-related deaths are estimated worldwide, [3] and approximately 34,000 patients are diagnosed, representing about 3% of all newly diagnosed cancers in the United States [4]. Moreover, the OSCC mortality rate is 3.7 per 100,000 in Japan [5]. OSCC has a high potential for local invasion and nodal metastasis and over 80% of early stage OSCC patients can be rescued by treatment, whereas less than 70% of advanced stage OSCC patients are incurable. The overall 5-year survival rates of OSCC have not improved significantly in the past 30 years, and it remains less than 50% [6]–[8]. Therefore, early detection and elucidation of the detailed molecular mechanism of OSCC are important.

Prospero homeobox 1 (Prox1) is a mammalian homologue of the Drosophila homeobox protein, prospero [9]. Prox1 is important for the embryonic development of the central nervous system, heart, lymphatic system, skeletal muscles, lens, retina, liver, pancreas, and kidney [10], [11]. Prox1 acts as a tumor suppressor in hematologic malignancies [12], esophageal cancer [13], hepatoma [14], pancreatic cancer [15], [16], breast cancer [17], and carcinomas of the biliary system [18]. However, recent reports have demonstrated that upregulation of Prox1 is a predictor of poor outcome in colon cancer [11], [19], glioma [10], and many vascular endothelial tumors [20], [21]. Prox1 is suggested to play various tissue-dependent functional roles, which reflect both an oncogenic potential and a tumor-suppressive role [22]. Thus, the role of Prox1 in malignancies remains controversial.

The forkhead box (FOX) transcription factors are a large family of proteins with similar DNA-binding domains [23], [24]. Expression of FOXC2 protein was detected in a majority of breast adenocarcinomas, including lobular and ductal adenocarcinomas, and colon adenocarcinoma [25], [26]. FOXC2 expression has also been reported in esophageal cancer and could be used as a novel independent prognosis factor [27]. FOXC2 is also an important regulator of epithelial to mesenchymal transition (EMT) in cancer cells [28], while the role of FOXC2 in oral squamous cell carcinoma (OSCC) remains unknown.

Angiogenesis and lymphangiogenesis are pivotal for tumor progression and nodal metastasis [29]. The major angiogeneic and lymphangiogenic factors are the vascular endothelial growth factor (VEGF)-A and VEGF receptor (VEGFR) 2 and the VEGF-C/−D and VEGFR3 systems, respectively [30], [31]. We previously reported that the VEGF family promotes tumor progression and nodal metastasis by inducing angiogenesis and/or lymphangiogenesis in OSCC [2], [29]. More recently, Prox1 was shown to induce lymphangiogenesis by activating VEGFR3 [32]. Prox1 is also a marker for lymphatic endothelial cells [33]. FOXC2 is a regulator of angiogenesis [26] and lymphangiogenesis [34]. Furthermore, Prox1 and FOXC2 are co-expressed and required for the onset of lymphovenous valve formation [35]. In the present study, we examined the expression and role of Prox1 and FOXC2 in human OSCCs.

## Materials and Methods

### Surgical Specimens

Formalin-fixed, paraffin-embedded 163 cases of primary OSCCs (89 men and 74 women, Age range, 44–91 years; means, 66.7 years) were used. We also utilized 15 frozen samples of OSCC (9 men and 6 women, Age range, 52–79 years; means, 65.8 years) and 5 cases of non-tumor oral mucosa (3 men and 2 women, Age rage, 36–52 years; means, 45.2 years) for gene expression analysis of Prox1 and FOXC2. All specimens were randomly selected from Nara Medical University Hospital, Kashihara, Japan. All cases were performed without pre-operative therapy. Tumor staging and the histological grade of OSCCs were classified in order to UICC TNM classification system, 7th edition and WHO criteria, respectively.

### Ethics Statement

This study was approved by the Medical Ethical Committee of the Nara Medical University (approval number. 719). Medical records and prognostic follow-up data were obtained from the patient database maintained by the hospital. The study protocol using human samples were performed according to the ethical standards expressed in the Declaration of Helsinki. Written informed consent was obtained from individual patients for use of their tissue samples. For strict privacy protection, identifying information for all samples was removed before analysis.

### Immunohistochemistry

Consecutive 3 μm sections were cut from each block, and immunohistochemistry was performed as we described previously. An immunoperoxidase technique was done following antigen retrieval with microwave treatment (95°C) in citrate buffer (pH 6.0) for 45 min. After endogenous peroxidase block by 3% H_2_O_2_-methanol for 15 min, specimens were rinsed with phosphate-buffered saline (PBS) three times. Anti-Prox1 antibody (Santa Cruz Biotechnology, Inc., Santa Cruz, CA, USA), anti-FOXC2 antibody (Santa Cruz Biotechnology), anti-CD34 antibody (a marker for vascular endothelial cells) (DAKO, Carpinteria, CA, USA), and anti-LYVE-1 antibody (a marker for lymphovascular endothelial cells) (Abcam, Tokyo, Japan) diluted by 0.5 μg/ml were used for primary antibody. After 2 h incubated at room temperature, specimens were rinsed with PBS three times and treated for an hour at room temperature with the secondary antibody peroxidase-conjugated anti-mouse (Medical & Biological Laboratories Co., Ltd., Nagoya, Japan) diluted at 0.5%. The specimens were then rinsed with PBS three times and color-developed with diaminobenzidine (DAB) solution (DAKO). After washing, specimens were counterstained with Meyer’s-hematoxylin (Sigma Chemical Co., St. Louis, MO, USA). Immunostaining of all samples was performed at the same conditions of antibody reaction and DAB exposure.

### Evaluation of Immunohistochemistry

Immunoreactivity of Prox1 and FOXC2 were classified according to Allred’s score (AS) [36] and we divided the immunoreactivity into 4 grades by AS; Grade 0, AS is 0; Grade 1, AS is 2 ∼ 4; Grade 2, AS is 5, 6; Grade 3, AS is 7, 8. Cases with Grade 2 and 3 were regarded as immunologically positive [2]. The microvessel density (MVD) and lymphovessel density (LVD) were measured on anti-CD34 and anti-LYVE-1 antibody immunopositive specimens, respectively. To quantify MVD or LVD, 5 maximum vessel density fields were selected from around of the tumor cells (the ‘hot spot’) and examined under a 200-fold magnification by microscope and averaged. We divided the tissue samples into two groups according to MVD levels; those with values higher than the mean value for the entire group, and those with lower than the group mean value. The same was applied based on the LVD values [29].

### Cell Culture

Human OSCC cell lines, KON, HSC2, HSC3, HSC4, Ca9-22 and SAT cells were obtained from Health Science Research Resources Bank and maintained in Dulbbeco’s modified Eagle’s medium (DMEM) (Wako Pure Chemical industries, Ltd., Osaka, Japan) supplemented with 10% fetal bovine serum (FBS) (Sigma Chemical Co., St. Louis, MO, USA) under the conditions of 5% CO_2_ in air at 37°C. KON and HSC3cells have high metastatic potential, HSC4 cells have low metastatic ability, and HSC2, Ca9-22, and SAT cells have no ability of metastasis and invasion. Primary human umbilical vein endothelial cells (HUVECs) and primary human dermal lymphatic microvascular endothelial cells (HDLMVECs) were purchased from Cell Applications (San Diego, CA, USA) and maintained in Endothelial growth media (Cell Applications) and Microvacular endothelial growth media (Cell Applications) under the conditions of 5% CO_2_ in air at 37°C, respectively.

### Quantitative Reverse Transcription-polymerase Chain Reaction

Total RNA was extracted using RNeasy Mini Kit (Qiagen Inc., Valencia, CA, USA) and total RNA (1 μg) was synthesized with the ReverTra Ace qRT Kit (Toyobo, Osaka, Japan). Quantitative reverse transcription-polymerase chain reaction (qRT-PCR) were performed on StepOne Plus Real-Time PCR Systems (Applied Biosystems, Foster City, CA, USA) using TaqMan Fast Universal PCR Master Mix (Applied Biosystems) and analyze the relative standard curve quantification method. PCR condition was according to the manufacturer’s instructions and GAPDH mRNA level was amplified for internal control. TaqMan Gene Expression Assays of Prox1, FOXC2, VEGF-A, VEGF-C, VEGF-D, and GAPDH were purchased from Applied Biosystems. All PCRs were done at triplicate.

### Small Interfering RNA

Stealth Select RNAi (siRNA) for Prox1 (HSS108597) and FOXC2 (HSS142054) was purchased from Invitrogen (Carlsbad, CA, USA). AllStars Negative Control siRNA (catalog No. 1027281) was used for control (Qiagen Inc). Twenty-nM siRNA were transfected with Lipofectamine 2000 (Invitrogen) according to the provider’s protocol.

### Cell Growth Assay

The cells were seeded at density of 2,000 cells per well of 96-well tissue culture plates and incubated for 48 h at 37°C. Cell growth was assessed by MTT assay using the incorporation of 3-(4,5-dimethylthiazol-2-yl)-2,5-diphenyltetrazolium bromide (Sigma Chemical Co.). The experiments were performed in triplicate.

### *In vitro* Invasion Assay

A modified Boyden chamber assay was done using the BD BioCoat Cell Culture Inserts glued to type IV collagen (Becton-Dickinson Labware, Bedford, MA, USA) as described previously. Briefly, cells were suspended in 500 μl of DMEM and placed in the insert. After 48 h incubation at 37°C, the filters were stained with hematoxylin. The stained cells were counted in whole inserts at 100×magnification. Each experiment was repeated at least three times.

### Cell Growth of Endothelial Cells Treated with Conditioned Medium from OSCC Cells

To generate conditioned media, 1×10^5^ negative siRNA, Prox1 siRNA, or FOXC2 siRNA treated KON cells were seeded in 24-well plates. After 24 h incubated at 37°C, the culture media were collected and centrifuged at 1,500 rpm for 5 min to remove pellet and collected the supernatants. HUVECs and HDLMVECs were seeded at a density of 2,000 cells in 96-well plates and incubated to overnight. Cells were then cultured with only Endothelial growth media, Microvacular endothelial growth media, or conditioned media (negative siRNA, Prox1 siRNA, or FOXC2 siRNA treatetd KON media and Endothelial growth media or Microvacular endothelial growth media (1∶1)). After 48 h, growth ability of endothelial cells was measured with the MTT assay [37].

### Migration of Endothelial Cells Co-cultured with OSCC Cells

The *in vitro* endothelial cell migration assay was performed using BD BioCoat endothelial cell Migration Assay System (Becton-Dickinson Labware) according to the provider’s manual. Briefly, 1×10^5^ negative siRNA, Prox1 siRNA, or FOXC2 siRNA treated KON cells were seeded in a 24-well plate. After overnight incubated at 37°C, 5×10^4^ cells of HUVECs or HDLMVECs were seeded in fibronectin pre-coated transwell chambers, consisting of polycarbonate membranes with 8 μm pores and then placed in the 24-well plates. After 24 h incubation, migrating cells were fluorescent labeling and measured intensity of fluorescence.

### Statistical Analysis

Statistical analysis was carried out with JMP8 (SAS Institute, Cary, NC, USA). Statistical differentiation was calculated with χ^2^ test, one-factor ANOVA test, and student-t test. Disease-free survival was analyzed by the Kaplan-Meier method, and differences between groups were calculated by means of a logrank test. Univariate analysis for disease free survival was calculated by logrank test. For multivariate analysis, Cox proportional hazards model was used (described as hazard ratio with 95% confidence intervals [95% CI], together with the P value). P values less than 0.05 were regarded as statistically significant.

## Results

### Expression of Prox1 and FOXC2 in Human OSCC Specimens

**Figure 1:**
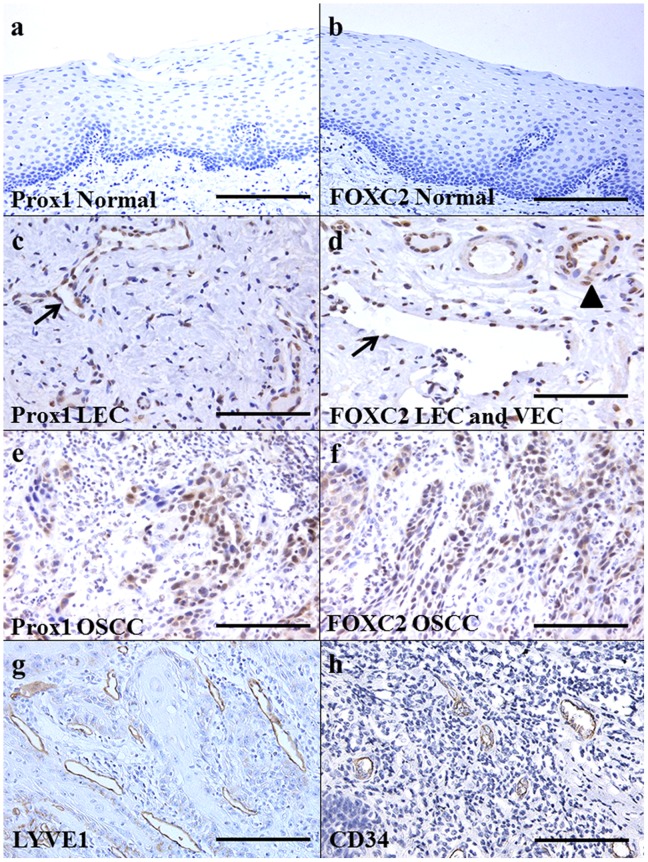
Immunohistochemical analysis of Prox1 and FOXC2 in human OSCC cases. Prox1 (a) and FOXC2 (b) expression were not observed in non-tumor oral mucosa. The lymphoendothelial cells (arrow) showed immunoreactivity to Prox1 (c) and expression of FOXC2 was found in lymphoendothelial cells (arrow) and vascular endothelial cells (arrow head) (d). Expression of Prox1 (e) and FOXC2 (f) were observed in cytoplasm of the cancer cells. LYVE1 positive lymphovessels (g) and CD34 positive blood vessels were counted for MVD and LVD, respectively. Original magnification was 200-fold. Bar, 100 μm. LEC; lymphoendothelial cells, VEC; vascular endothelial cells.

First, we examined the expression of Prox1 and FOXC2 in human OSCCs by immunohistochemistry. Although non-cancerous oral mucosa did not express Prox1 (Fig. 1a) and FOXC2 (Fig. 1b), immunopositivity of Prox1 was observed in lymphoendothelial cells (LECs) (Fig. 1c) and expression of FOXC2 was observed in LECs and vascular endothelial cells (VECs) (Fig. 1d). Nuclear staining for these factors was observed in OSCCs, and 25.8% (42/163) and 23.3% (38/163) of OSCCs were positive for Prox1 (Fig. 1e) and FOXC2 (Fig. 1f), respectively. The relationship between the expression of Prox1 and FOXC2 and the clinicopathological characteristics of the OSCC specimens are summarized in Table 1. Immunoreactivity for Prox1 was observed in 62% (31/50) of the nodal metastasis-positive cases, whereas 9.7% (11/113) of the cases without nodal metastasis expressed Prox1 (P<0.0001). Prox1 expression was also associated with local progression of the tumor (T classification) (P = 0.0023), clinical stage (P<0.0001), and lymphatic vessel density (LVD) (Fig. 1g) (P<0.0001). No significant relationship was observed between the expression levels of Prox1 and age, sex, site, histological differentiation, or microvessel density (MVD). The expression of FOXC2 was associated with MVD alone (Fig. 1h) (P<0.0001). Elevated expression of Prox1 was observed to be correlated with the overexpression of FOXC2 in OSCCs (P<0.0001).

**Table 1:** Relationship between Prox1 or FOXC2 expression and clinicopathological parameters.

	Prox1		FOXC2	
Parameters	negative	positive	negative	positive
Gender				
Male	70	19	52	22
Female	51	23	73	16
P value	0.1572		0.0733	
Age				
<−65	53	15	52	16
>65	68	27	73	22
P value	0.3598		0.9559	
Site				
Tongue	64	21	66	19
Gingiva	34	16	36	14
Other	23	5	23	5
P value	0.3713		0.57	
Histologicaldifferentiation*				
Well	63	25	69	19
Moderately	44	14	42	16
Poorly	14	3	14	3
P value	0.6103		0.5934	
T classification				
T1	27	2	20	9
T2	60	16	59	17
T3	22	13	27	8
T4	12	11	19	4
P value	0.0023		0.6915	
Clinical stage				
I	26	2	20	8
II	52	7	47	12
III	27	16	34	10
IV	16	17	24	8
P value	<0.0001		0.8532	
Nodal metastasis				
Negative	102	11	89	24
Positive	19	31	36	14
P value	<0.0001		0.4129	
FOXC2				
Negative	100	25	–	–
Positive	21	17	–	–
P value	0.0049			
MVD**	39.2±25.7	40.8±29.3	19.3±10.1	41.2±32.2
P value	0.7380		<0.0001	
LVD**	13.9±10.5	31.5±25.2	27.2±13.7	29.3±19.3
P value	<0.0001		0.456	

Relationship between expression of Prox1 or FOXC2 and parameters excluding MVD and LVD were calculated by chi-square test. Relationship between expression of Prox1 or FOXC2 and MVD or LVD were calculated by one-factor ANOVA test. T classification and clinical stage were classified according to the TNM classification.

*Histological differentiation: Well, well-differentiated squamous cell carcinoma; Mod, moderately differentiated squamous cell carcinoma; Por, poorly differentiated squamous cell carcinoma.

**MVD and LVD were Means ± S.D. (standard deviation), each S.D. was less than 10% in all cases.

**Figure 2:**
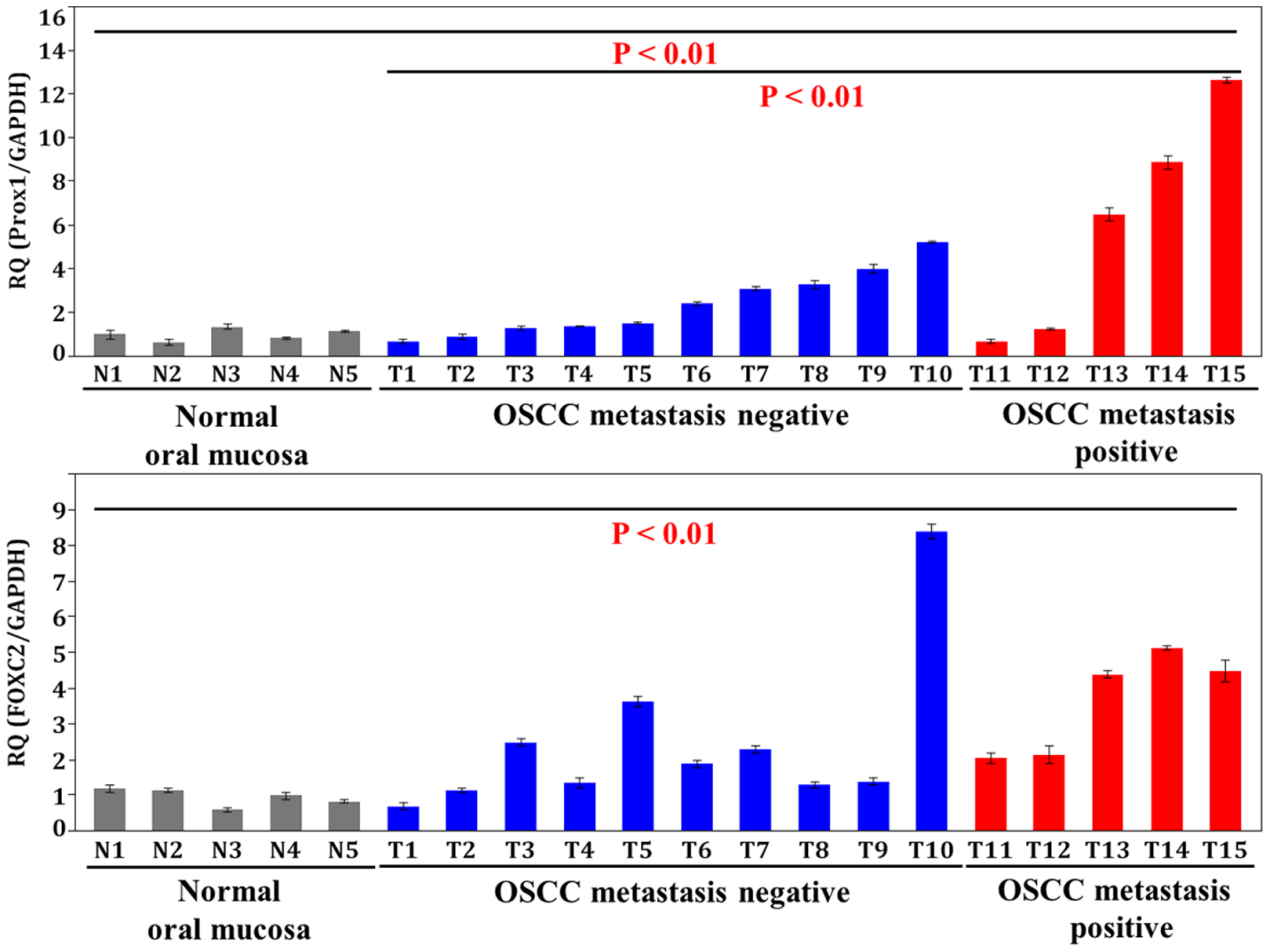
Gene expression of Prox1 and FOXC2 by qRT-PCR. The mRNA expression levels of Prox1 (P<0.01) and FOXC2 (P<0.01) in OSCCs were higher than normal oral mucosa. Prox1 expression was upregulated in nodal metastasis positive OSCCs than in those with negative OSCCs (P<0.01). GAPDH was used for internal control. Error bar, standard deviation (S.D.).

We then assessed the expression of Prox1 and FOXC2 mRNA in 5 samples of non-tumor oral mucosa, 10 samples of metastasis-negative OSCCs, and 5 samples of metastasis-positive OSCCs (Fig. 2). The expression of Prox1 (P<0.01) and FOXC2 (P<0.01) were higher in OSCCs than in the normal oral mucosa. Although Prox1 was upregulated in nodal metastasis-positive OSCCs unlike that in the metastasis-negative OSCCs (P<0.01), the expression levels of FOXC2 were not significantly different between the 2 OSCC groups.

### Relation between Prox1 and FOXC2 Expression and Prognosis of OSCCs

**Figure 3:**
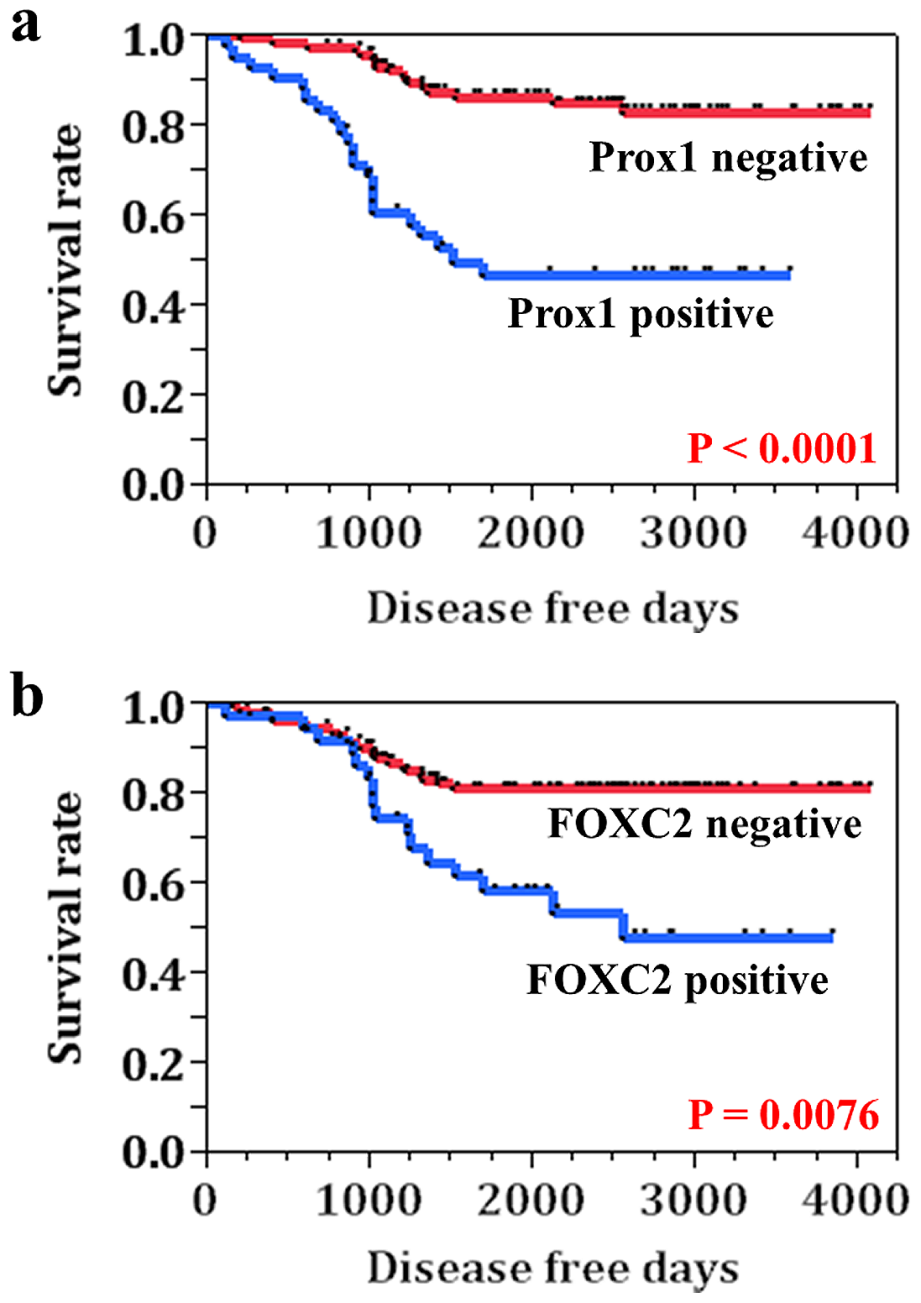
Disease free survival in OSCC cases. **a**, Prox1-positive cases had significantly reduced disease-free survival compared to the Prox1-negative patients (P<0.0001). **b**, The patients with FOXC2-positive tumors also had significantly poor prognosis than those with FOXC2-negative tumors (P = 0.0076).

Local and nodal recurrence occurred in 38 of the 163 patients whose tumor specimens were evaluated in this study. An analysis of disease-free survival showed that Prox1-positive patients had significantly reduced disease-free survival compared to the Prox1-negative patients (P<0.0001) (Fig. 3a). The patients with FOXC2-positive tumors also had significantly worse prognosis than those with FOXC2-negative tumors (P = 0.0076) (Fig. 3b). Univariate analysis performed using the log-rank test indicated that clinical stage (P = 0.0055), nodal metastasis (P = 0.0002), Prox1 expression (P<0.0001), and FOXC2 expression (P = 0.0018) were associated with poor outcome in OSCCs. Multivariate analysis performed using the Cox proportional hazards model showed that only Prox1 expression (P = 0.0039) was a prognostic factor for disease-free survival (Table 2).

**Table 2:** Univariate and multivariste analysis of disease free survival.

Parameters	P value		
Gender (F–M)	0.1550		
Age (<_65–>65)	0.3618		
Histology (well, mod, por)	0.5608		
Site (tongue, gingiva, other)	0.9446		
T factor (T1–4)	0.3938		
Stage (I–IV)	0.0055		
Nodal metastasis (negative - positive)	0.0002		
Prox1 (negative - positive)	<0.0001		
FOXC2 (negative - positive)	0.0076		
**Parameters**	**HR**	**95% CI**	**P value**
Clinical stage			
I	1.0000		
II	0.5194	0.1842–1.4863	0.2156
III	0.3475	0.0919–1.1987	0.0944
IV	0.7691	0.1905–2.9277	0.7032
Nodal metastasis			
Negative	1.0000		
Positive	1.6501	0.5234–5.7912	0.4032
Prox1			
Negative	1.0000		
Positive	3.3692	1.4770–7.6958	0.0039
FOXC2			
Negative	1.0000		
Positive	1.8986	0.9585–3.6893	0.0655

Univariate analysis was performed by log lank test. Multivariate analysis was calculated by means of Cox proportional hazard model. HR and 95% CI mean hazard ratio and 95% confidence intervals, respectively.

### In vitro Analysis of Prox1 and FOXC2 in OSCC Cells

**Figure 4:**
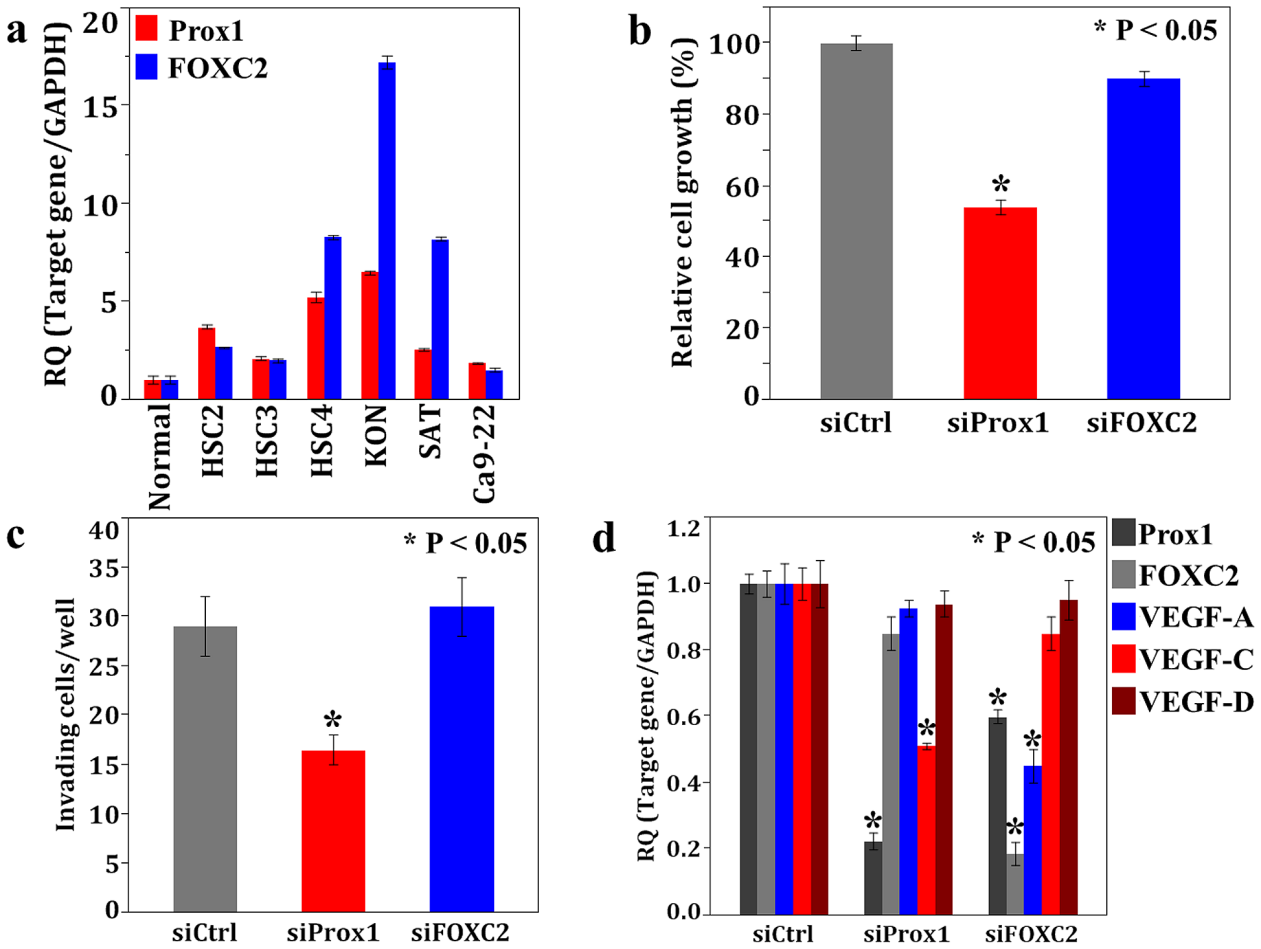
*In vitro* analysis of Prox1 and FOXC2 using human OSCC cells. **a**, Expression of Prox1 and FOXC2 in 6 OSCC cells measured using realtime RT-PCR. Highly metastatic KON cells showed higher expression of both genes. Expression levels of GAPDH was used for internal control. **b**–**d**, Effects of Prox1 or FOXC2 siRNA treatment in KON cells on cell growth (b), invasive ability (c),and mRNA expression levels of Prox1, FOXC2, VEGF-A, VEGF-C, VEGF-D (d). Growth and invasive ability were examined by the MTT assay and an in vitro invasion assay. Expression levels of Prox1 and VEGF-C were decreased by Prox1 siRNA treatment and decreases in FOXC2, Prox1, and VEGF-A expression were observed in FOXC2 siRNA treated KON cells (d).

We evaluated Prox1 and FOXC2 expression in cultured OSCC cells. Expression levels of Prox1 and FOXC2 in KON cells were higher than that in the other OSCC cell lines (Fig. 4a). To examine the effects of Prox1 and FOXC2 in OSCC, we further performed an in vitro analysis using KON cells. Cell growth and invasion potential of the KON cells treated with Prox1 siRNA was inhibited compared to that of the cells treated with the negative control siRNA. Knockdown of FOXC2 with siRNA did not affect the ability of KON cells to grow and invade (Fig. 4b, c).

Next, we verified the effects of Prox1 and FOXC2 on the expression of the genes encoding for the VEGF family in KON cells, because both factors were associated with MVD or LVD (Fig. 4d). Reduction of VEGF-C was observed following the knockdown of Prox1, whereas VEGF-A expression was attenuated upon treatment with FOXC2-specific siRNA in the KON cells. Furthermore, downregulation of Prox1 was observed in FOXC2 siRNA-treated KON cells. However, reduction of FOXC2 was not observed in KON cells treated with Prox1-specific siRNA. These results suggest that Prox1 was involved in regulating the expression of VEGF-C and that FOXC2 expression accelerated not only the activation of VEGF-A but also of the Prox1 in OSCC cells.

**Figure 5:**
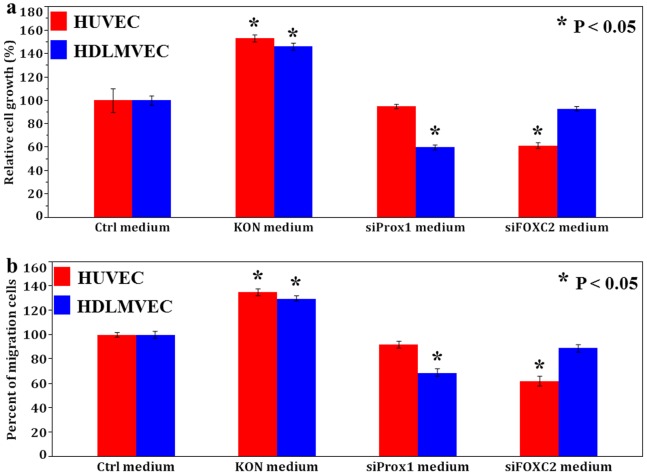
Cell growth and migration of endothelial cells affected by OSCC cells. **a**, Cell growth of endothelial cells treated with conditioned medium from OSCC cells. The growth of HUVECs and HDLMVECs were significantly enhanced by the addition of negative siRNA treated KON cell culture supernatant. Further, HUVECs or HDLMVECs proliferation was inhibited when added to FOXC2 or Prox1 siRNA treated KON cell culture supernatant, respectively. **b**, Migration of endothelial cells co-cultured with OSCC cells. The migration of HUVECs and HDLMVECs were enhanced by co-culture with negative siRNA treated KON cells. Moreover, co-cultivation with FOXC2 or Prox1 siRNA treated KON cells suppressed HUVECs or HDLMVECs migration, respectively.

Finally, we confirmed the influence of Prox1 and FOXC2 on angiogenesis and lymphangiogenesis in OSCC cells. The proliferation of HUVECs and HDLMVECs were significantly enhanced by the addition of culture supernatant derived from negative control siRNA-treated KON cell cultures, suggesting that the KON cells secreted cell growth-promoting factors. However, treatment of HUVECs or HDLMVECs with the culture supernatant from FOXC2- or Prox1-specific siRNA-treated KON cell cultures, respectively, resulted in the inhibition of cell growth (Fig. 5a). The migration of HUVECs and HDLMVECs was potentiated by co-culture with negative control siRNA-treated KON cells. However, co-culture with FOXC2- or Prox1-specific siRNA-treated KON cells significantly suppressed the migration of HUVECs or HDLMVECs, respectively, unlike hat observed in the cells that received the control treatment (Fig. 5b). These data pinpoint the roles of Prox1 and FOXC2, as regulators of lymphangiogenesis and angiogenesis in OSCC, respectively. In addition, we obtained similar results in other OSCC cells (data not shown).

## Discussion

In the present study, we found that the mRNA expression levels of Prox1 or FOXC2 in OSCCs were higher than that in the normal oral mucosa. By immunohistochemistical analysis, Prox1 was shown to be closely associated with tumor progression (T factor and clinical stage), nodal metastasis, and LVD, and FOXC2 expression was shown to be significantly correlated with MVD in OSCCs. The patients with Prox1- or FOXC2-positive OSCCs had shorter disease-free days, and Prox1 expression was an independent poor prognostic factor in OSCC. We also found that Prox1 accelerates migration, invasion, VEGF-C expression, and lymphovascular endothelial cell proliferation and migration in OSCC cells. Furthermore, FOXC2 was shown to regulate the expression of VEGF-A and Prox1 as well as the growth and migration of vascular endothelial cells. We also confirmed that Prox1 and FOXC2 induced tube formation in the endothelial cells (data not shown). We speculate that the interactions of Prox1 and VEGF-C are the cause of reduce growth and migration ability of HUVECs by Prox1 knockdown treatment (Fig. 4d, Fig. 5). Indeed, it has been defined tumor cells-secreted VEGF-C knockdown inhibits HUVECs proliferation and migration [38] and we also clarified VEGF-C is accelerates angiogenesis in OSCC [29]. We also infer that the reason for growth and migration potential of HDLMVECs reduce upon siRNA treatement of FOXC2 are FOXC2 regulates expression of Prox1 (Fig. 4d, Fig. 5).

Prox1 is a nuclear transcription factor [22] and is activated in colon cancer [11], [19], WHO grade II gliomas [10], and many vascular endothelial tumors [20], [21]. Prox1 has been reported to promote tumor progression by influencing cancer cell migration and invasion in colon cancer [19] and kaposiform hemangioendothelioma [20], and our results are consistent with these findings. Elsir et al indicate that enhanced expression of Prox1 in the context of activated Wnt/β-catenin signaling and loss of p53 function may be associated with oncogenesis [22]. On the other hand, Prox1 acts as a tumor suppressor in hematologic malignancies [12], esophageal cancer [13], hepatoma [14], pancreatic cancer [15], [16], breast cancer [17], and carcinomas of the biliary system [18]. It has been revealed that expression of Angiopoietin 2 (Ang2) is enhanced by Prox1 in endothelial cells [39]. Further, Ang2 is one of the important regulators of angiogenesis and lymphangiogenesis and correlated with poor prognosis in OSCC [40]. Prox1 may play a critical role in the Ang2-induced lymphangiogenesis and angiogenesis of OSCC. Further functional and large scale clinicopathological examinations using various series of cancers are warranted in order to clarify the roles of Prox1 in cancers.

FOXC2 is an oncogene in breast cancer [25], colon cancer [26], and esophageal cancer [27]. Although FOXC2 protein has a cytoplasmic localization in cancer cells [25]–[27], our immunohistochemistry results showed that FOXC2 is detectable in the nuclei of cancer cells. We also verified that FOXC2 protein cannot be detected by immunoblotting using a fraction containing extranuclear proteins extracted from OSCC cells (data not shown). FOXC2 protein may be transported outside the nucleus in certain types of cancers, and further studies will be needed to delineate the localization of FOXC2 in various cancers. FOXC2 is also one of the key players in the epithelial-to-mesenchymal transition (EMT) [28] and EMT inducers, such as TGF-β, stimulate FOXC2 expression in cancer cells [24]. FOXC2 represses and activates E-cadherin and vimentin expression, respectively [24], [28]. We also ascertained that FOXC2 regulates the expression of some EMT-related markers in OSCC cells (unpublished data); however, further studies are required to ascertain the relationship between FOXC2 and EMT in OSCC.

Angiogenesis plays a critical role in prenatal development, wound healing, chronic inflammation, tumor progression, and metastasis, and lymphangiogenesis promotes growth and nodal metastasis in cancer cells [41]. Prox1 is a regulator of lymphangiogenesis during prenatal development and inflammation through the upregulation of VEGFR3 [22], [32], and we also confirmed that Prox1 promotes lymphangiogenesis by activating VEGF-C, the product of which is one of the ligands of VEGFR3, in OSCCs. However, a direct role for Prox1 in angiogenesis has not been established yet. A previous report showed that Prox1 transformed blood endothelial cells to lymphatic endothelial cells [42]. Prox1 produced by cancer cells may also trigger lymphangiogenesis from lymphatic and blood endothelial cells in OSCCs. FOXC2 is pivotal for the migration and tubular transformation of vascular endothelial cells and for tumor angiogenesis, functions that are elicited by the activation of VEGF-A signaling [24], [26]. Although FOXC2 is also expressed in lymphatic endothelial cells, the contribution of FOXC2 to lymphangiogenesis in malignancies remains unknown [24], [34]. We determined that FOXC2 induces tumor angiogenesis through VEGF-A, but that it is not involved in lymphangiogenesis in OSCCs, results that are somewhat in accord with past reports. It is generally accepted that angiogenesis and/or lymphangiogenesis promote tumor progression in malignancies [41], and we previously reported that VEGF family-mediated angiogenesis and/or lymphangiogenesis are associated with tumor progression, nodal metastasis, and worse prognosis in OSCCs [2], [29]. On the other hand, it has been suggested that angiogenesis and lymphangiogenesis do not necessarily promote tumor progression. Previously reports have been indicated that VEGF-A is not associated with angiogenesis and that VEGF-C/D are not associated with nodal metastasis in cancer [43]–[46]. It has also been reported that MVD and LVD have no effect on tumor progression [47], [48]. Therefore, the role of angiogenesis and lymphangiogenesis in tumor progression remains controversial. In vivo studies will be helpful in the future to further clarify the role of tumor angiogenesis and lymphangiogenesis in malignancies.

In conclusion, the present study demonstrates that Prox1 and FOXC2 act as oncogenes by inducing lymphangiogenesis and angiogenesis in OSCC, respectively. Moreover, we found that FOXC2 is involved in the regulation of Prox1 expression. Further investigation of Prox1 and FOXC2 expression and function may offer additional insights for the diagnosis and treatment of human OSCCs.
